# Circular of the National Institute

**Published:** 1846-10

**Authors:** James Wynne

**Affiliations:** Chairman Committee; Baltimore


### CIRCULAR OF THE NATIONAL INSTITUTE.

The following circular of the National Institute at Washington ought to elicit valuable information, and we do what we can to give it publicity:

Baltimore, 20th July, 1846.

Sir:—The Medical Department of the National Institute at Washington directed a committee of its members to institute an inquiry into the sanatory condition of the United States, and report the result of its labors to that body. In order to facilitate this important enquiry, you will greatly oblige the committee by an answer to the following enquiries:

1st. What is the medical topography of your district, and the influence of its soil and climate over health and disease?

2d. What has been the effect of agriculture, the clearing of forests, and the draining of soils, upon the climate and health of the inhabitants?

3d. What manufactories or large towns are there in your district, and what is their effect over the health of those exposed to their influence?

4th. What evils operate in the towns requiring municipal interference? Are they deficient in a supply of water, drainage, or cleanliness, and how remedied?

5th. In towns what amount of population, and what provision for public squares?

6th. What epidemic or endemic diseases have you observed, and to what cause are they to be attributed?

7th. What is the annual number of deaths, births, and marriages to each thousand of your population? Transmit your bills of mortality, if any.

8th. Are there any remarkable instances of longevity, and what is their age, and mode of life?

9th. Are there any retreats peculiarly favorable to invalids affected with pulmonary complaints, or any medical springs, and their qualities and effects?

Be pleasedto prepare your reply in such form as you desire it to be printed in the committee's report, and direct it to the undersigned, under cover from your member of Congress. Your immediate attention is requested. Respectfully, your obedient servant,

JAMES WYNNE, M. D., Chairman Committee.

Prof. Sewell, of Columbia College; Prof. Thomas, do. do.; Dr. Buck, U. S. Army; Dr. Washington, U. S. Navy; Dr. Wynne, members of the committee.

